# Ask me what is in my heart of hearts! The core question of care in relation to parents who are patients in a psychiatric care context

**DOI:** 10.3402/qhw.v11.30758

**Published:** 2016-06-23

**Authors:** Nina Elisabeth Blegen, Katie Eriksson, Terese Bondas

**Affiliations:** 1 Department of Nursing, Faculty of Health Sciences, Oslo and Akershus University College of Applied Sciences, Oslo, Norway; 2 Department of Caring Science, Åbo Academy University, Vaasa, Finland; 3 Faculty of Professional Studies, Nord University, Bodø, Norway

**Keywords:** Caritative caring, caring science, ethics, hermeneutics, mothers, patient, psychiatric care, responsibility

## Abstract

The aim is to understand the experience of being cared for in psychiatric care as a patient and as a parent. Parenthood represents the natural form of human caring, a human directedness regardless of gender. The study has its starting point in this image, as it applies to mothers who receive care as provided in a psychiatric care context. The theoretical perspective is the theory of caritative caring, and the methodological approach is the philosophical hermeneutics outlined by Gadamer. The sample was purposeful: 10 mothers who experienced being a mother while suffering from mental illness and receiving care from professionals in psychiatric specialist health care contexts. The interpretation process is inductive, deductive, and abductive, and includes different levels of rational, contextual, existential, and ontological interpretation supported by the chosen theoretical perspective and the philosophy of ethics outlined by Emmanuel Levinas. The interpretation on the contextual level shows that the patients do not talk about their inner feelings concerning themselves as mothers in the care relationship. The interpretation on the existential level reveals the meaning of the mothers’ experiences of inner struggle between their inner demands and assuming a mask of silence. The patients’ experiences on the ontological level were interpreted as a struggle between the responsibility inherent in human being and the fear of condemnation. At the ontological level, a new hypothesis of the understanding of the meaning of the parents’ experiences was formulated: Being in care as a patient and as a parent means struggling to restore one's responsibility as a human being. This new understanding paves the way for caring of the patient who is a parent.

The aim of this study is to understand the patients’ experiences of being in care provided in a psychiatric care context. The purpose is to understand the patients’ inner world in health and suffering, when being in care, and the scientific interest is directed towards understanding the core question of care related to patients who are parents in psychiatric care. *The patient* is, in this study, understood as *the suffering human being* (Eriksson, 1990, 1997b, 2001, 2006; Lindström, Lindholm, & Zetterlund, 2010). The present study is a part of a larger study and only female subjects are included, but it is the phenomenon of *care and caring* as an existential and ontological dimension in human beings that is in focus. Caring acts are natural processes essential for human survival, growth, and development, reflecting a human directedness regardless of natural gender (Eriksson, 1997b, 2001; Levinas, 2013; Lindström et al., 2010). All forms of parenting, mothering or fathering, involve relationships characterized by responsibility and caring for a certain child or certain children, where a person and a child are in a continuous, mutual, and changeable care relationship, independent of biology, context, culture, and social norms (Holm, 1993; Levinas, 1990; Ruddick, 2002). Caring acts are expressed in a number of ways depending on circumstances, time, and place, but the core of caring—the responsibility, compassion, and human love—is general and eternal (Eriksson, 1990, 1997a, b; Levinas, 1990). Caring in its original sense implies a relationship between self and others, in which hope, love, and charity are conveyed through tending, playing, and learning (Lindström et al., 2010). Parenthood involves taking on responsibilities that extend beyond one's own life, because the child represents possibilities in a yet unknown future (Levinas, 1990). Mothers and fathers understand their child's life and destiny as parts of their own life (Levinas, 1990, 2013); caring acts in parenting are accordingly inseparably connected to human beings’ existential and ontological lives (Bondas, 1998, 2000; Bondas & Eriksson, 2001).

Research has shown that parents seek help not only for themselves but for the sake of their children (Blegen, Eriksson, & Bondas, 2014; Blegen, Hummelvoll, & Severinsson, 2012; Ende, Busschbach, Nicholson, Korevaar, & Weghel, 2016; Montgomery, Mossey, Adams, & Bailey, 2012; Montgomery, Mossey, Bailey, & Forchuk, 2011; Tjoflåt & Ramvi, 2016). Studies on mothers and fathers with mental illnesses describe experiences of being neglected and/or denounced in psychiatric contexts (Blundell, Wittkowski, & Hare, 2012; Ende et al., 2016; Montgomery et al., 2011; Price-Robertson, Reupert, & Maybery, 2015; Styron, Pruett, McMahon, & Davidson, 2002). Patients’ narratives about their suffering report a deep existential struggle (Lindström & Lindholm, 2003; Nilsson, Nåden, & Lindström, 2008). Suffering from mental illness is often experienced as being fragmented and flawed, as if being a stranger to one's inner world (cf. Lassenius, 2005; Nilsson, 2008; Power, Jackson, Weaver, & Carter, 2011). Many therefore keep their inner feelings and thoughts to themselves out of fear of being stigmatized and losing custody of their children (Byatt, Biebel, Friedman, Debordes-Jackson, & Ziedonis, 2013; Lacey et al., 2015; Montgomery et al., 2011; Rørtveit, Åstrøm, & Severinsson, 2009; Rørtveit, Åstrøm, & Severinsson, 2010). In suffering, the human being strives for integrity and wholeness, and when one's inner strength comes to a loss, we search out professional care to reconcile ourselves to the conditions of life (Eriksson, 2000, 2006).

The idea is that the context of psychiatric care constitutes both a dimension of external limits concerning caring culture, environment, and circumstances, and an inner dimension of meaning concerning the eternal existential and ontological questions in life (Eriksson, 2001; Kasén, 2002; Lindström & Lindholm, 2003).

The strong focus on effective and evidence-based practice in health and nursing care and research involves a risk of losing sight of important ethical values related to care and caring culture. Hence, health care in general miss out on important discussions regarding the human being's inner responsibility and moral virtue as it is incorporated in their existential and ontological world. Avoiding the human being's existential dimension and its fundamental ontological condition might lead to insufficient understanding of the patient's world, as well as of the inner world of the professional carer. We need to develop our knowledge of caring in a manner that includes an existential and ontological basis for patients who are parents and are cared for in a psychiatric care context. We also need to develop the theory that supports this type of understanding, and in this study the theory of caritative caring was chosen (Eriksson, 1990, 2001; Lindström et al., 2010). The theoretical perspective is both a starting point for the study, and basis for developing the theory of caritative caring. The study seeks to find new patterns in the core of caring through an explorative and disclosing abduction.

## Aim

The aim is to understand the experience of being cared for in psychiatric care when being a patient and a parent. The research question is: What are the experiences of being cared for in psychiatric care when being a patient and a parent?

## Theoretical perspective

The study is grounded in the humanistic tradition of caring science (Eriksson, 1990, 1997a, b, 2000, 2001, 2003, 2006, 2007; Lindström et al. 2010). The caring science is defined by its ontology and human orientation. Caring is something human by nature, and is expressed through tending, playing and learning in order to attain integrity and wholeness that is compatible with bearable suffering (Eriksson, 1997b, 2007; Lindström et al., 2010). Tending represents concrete qualitative characteristics such as a feeling of being welcome, confidence, hope, and safety. Playing includes the characteristic hallmarks of spontaneity, imagination, creativity, desires, and wishes; while learning encompasses development, constant growth, and change (Eriksson, 1997b; Lindberg, von Post, & Eriksson, 2013). Caring is ontological and an expression of caritas; it is the innermost being of caring that we search coming to the fore. Professional caring is based on the natural caring and is constituted by the idea of motherliness (Eriksson, 1990; Levinas, 1990), which implies the genuine, universal, spontaneous and unconditional love and charity that characterizes caritative caring (Lindström et al., 2010).

The human being is seen as an integrated entity of body, soul, and spirit, where health and suffering are prevailing conditions in life. Health consists of movements between the actual and the potential in the human being's process of becoming, where suffering is seen as a basic category of caring. Suffering has many faces and characteristics, has no distinct reason or definition, and is in lack of an explicit language. Suffering has no meaning, but by reconciliation meaning can be ascribed to it. Through reconciliation the feeling of wholeness and holiness is re-created. Ontologically, every human being is fundamentally seen as longing for and striving for wholeness and integration (Lindström et al., 2010). The inner world of the human being contains an inner space of existential and ontological dimensions (Lassenius, 2005), where suffering might unfold a desire and a longing for a deeper meaning in life (Ueland, 2013). Truly understanding the suffering human being's greater meaning and motive in health and suffering is to understand and respect the inherent human dignity (Eriksson, 2007). The goal of caritative caring is to restore the human being so that she can find her inner love and freedom and go back to her human mission: the responsibility to care and exist for the sake of others (Edlund, 2002; Eriksson, 2001; Lindström et al., 2010).

## Methodological approach

The hermeneutic philosophy of understanding as outlined by Hans Georg Gadamer (2006) has been the overall methodology and has been accompanying the interpretation process at every stage as an attitude. The focus of the research is to understand the human being's existential and the ontological world, which presupposes an epistemology that embraces the human being's ontology (cf. Gadamer, 2006). The ontological, fundamental conditions in hermeneutic philosophy are the reflection on the reality that goes beyond the immediate and visible world (Eriksson, 2010; Gadamer, 2006). The main objective is understanding, not as a method or a possession but as a being that constitutes itself through interpretation and understanding (Gadamer, 2006). According to Gadamer (2006), interpretation and understanding are ontology and cannot be seen as method, but must be seen as substance. Hence, the hermeneutic interpretation can take us deeper into the life world of the patient. To understand the subject matter for the patient means to have an open mind for the unfamiliar in the interview as well as to the interview text. An open minded attitude allows the subject matter coming forward and question the researchers' prior understanding. The basic element in hermeneutical studies is the pre-understanding, which is not unbiased (Regan, 2012). The researchers all have a pre-understanding of the sensitive issues associated with being a parent who suffers from mental illness, as well as an understanding formed by the experiential and practical knowledge gained through their professional experiences as nurses, mental health nurse, and researchers.

The sense of wholeness presupposes an understanding of the phenomenon based on a movement between the known and the unknown, between the parts and the whole; i.e., between the researcher's pre-understanding, the perspective of the patient from the empirical material, the caring science perspective, and the texts of philosophy. The idea is that there is a common and true reality beyond what is immediately visible, so the substance of the patient's experiences assigns itself in reality and is to be found in its natural form (Eriksson, 2010; Martinsen & Eriksson, 2009).

### The interpretation process

The interpretation process is a hermeneutical and a dialectical process moving between the researchers’ pre-understanding, the empirical material, and the chosen theory, and it consists of three phases: the inductive, the deductive, and the abductive. The phases involve different levels of abstractions. The inductive and deductive phases involve the rational, contextual, and existential level, which is inspired by Ödman (1992, 1997, 2007). The abductive phase includes the ontological level and is Råholm's (2010) and Peirce's (1990) understanding of scientific abduction. Ödman's model of interpretation of the human being's reality through a profound interpretation of the human existential conditions and possibilities, while the scientific abduction methodology developed by Peirce, and Råholm, is the basis for the reasoning of what might be the meaning of the participants’ experiences.

The interpretation process is affected by the researchers’ values, pre-understanding, and prejudices (Gadamer, 2006; Regan, 2012), but the hermeneutical reading, reflections, and the logical reasoning took into account the philosophy that guided the interpretation process at all levels. The hermeneutical interpretation process demands unprejudiced openness, awareness, careful use of theory, and search for the otherness of a phenomenon that is not given by one's pre-understanding (Dahlberg, Dahlberg, & Nystrøm, 2008; Gadamer, 2006; Regan, 2012).

The interpretation consists of four levels of abstractions: the rational level and the contextual level, which is close to the empirical material, and the existential and the ontological level, which is close to theory.

The understanding is not a result of detailed examination of the elements or the words of the text, but a result of reading, re-reading with questioning eyes and ears, reflection, and pensiveness. The known and the unknown meet through the reading: at the same time it is impossible not to read into the text what one already knows, but by assuming a painstaking and conscientious attitude, one can try to authorize the participant experiences. Abductive reasoning is a creative inference aimed at exploring an understanding of the subject matter, and involves three phases of reasoning: induction, deduction, and abduction (Table I).

**Table I:** The dialectical movements in the interpretation of the meaning of the mothers’ experiences of being cared for in psychiatric care when being a patient and parent.

Ontological level	Being in care as patient and mother means struggling to restore one's responsibility as a human being	Abductive
Existential level	Between the silent mask and the beating heart	Deductive
Contextual level	Dare I say it? The anxiety of disclose their inner world	
Rational level	“I don't manage to be myself in the meeting; I restrain myself even if I have something important on my mind. I do not dare to say it. Sometimes I am really scared and decide to talk about it, but when I arrive they ask me about how things are going and something else, so I leave without saying what I intended to say.” “I was afraid to talk. And they didn't ask, either, so I didn't want to say anything; instead, I focused on the improvement since I was afraid that the stuff about *diagnosis*, that it …, that I would be accused, or …” “My children were on my mind the whole time. Poor them; they haven't chosen their mommy. I cannot be ill to them; I just wanted to be there for them, but no one understood, so …”	Inductive

The first inductive phase starts from the researcher's pre-understanding and previous research and includes the descriptions of the participants’ narratives, the interview, the transcriptions, and the interview text, i.e., the rational level. The interview text was approached with an open and dialectical attitude in order to acquire an understanding of the mothers’ descriptions of their experiences of being in a caring relationship in a psychiatric context. The inductive phase was completed with an abstraction and description based on the researchers’ pre-understanding, previous research, and the interpretations of the mothers’ narratives, which constitutes the contextual level. The contextual level includes interpretation and descriptions of acts, thoughts, and feelings related to the experiences of being in care in the psychiatric context.

The second phase, the deductive, has its point of departure in the theory of caring science, and comprises existential assumptions based on previous interpretation and abstraction; that is, the existential level. Through an open and sensitive reflection on the relationship between the pre-understandings, the text, and the theory—through questioning not only the text but also the pre-understanding—at this stage the understanding went beyond the pre-understanding. The meaning that break through the interpretation process challenges and demands not only the researchers’ pre-understanding and prejudices, but also their existence and experiences in life (Gadamer, 2006; Regan, 2012). Levinas (2013) describes this as “what is said in the spoken words,” and associate it both to the individual and to the universal dimension in the human being. In the reading at this level it is possible to see the unknown, but also to ignore and overlook (Cavalcante Schuback, 2011; Gadamer, 2006). The interpretation at the deductive stage moves from the individual and particular to the general and universal. An open and dialectical approach enables receptiveness to letting the text speak and to questioning the researcher's pre-understanding. The pre-understanding became most highly visible at this stage of the interpretation process. In open dialogues between the theory and the interview text, common hallmarks of existential meaning related to the experiences of being in a care relationship emerged and were marked.

The abstraction at this level deals with existential feelings and experiences, for example fear, anxiety, guilt, and shame.

The third phase, the abduction, describes the potential or the possibilities, and is close to the ontological question of the human's being, its meaning and assumptions (Råholm, 2010). The interpretation went into dialectical movements between the interview text, previous understanding, and the chosen theory in order to grasp the ontological meaning of the mothers’ experiences; i.e., the ontological level. The interpretation process went back and forth between the pre-understanding, the empirical data, the theoretical perspective, and the chosen theory, from which theoretically charged empirics emerged. Ideas and assumptions about the ontological meaning of the mothers’ experiences were questioned and tested against the empirical material. The interpretation on the ontological level concerns the ethical meaning of the mothers’ experiences, i.e., how the human being constitutes herself through the responsibility inherent in caritative caring. The reasoning in this chapter reflects a comprehensive understanding that is based on interpretations and understanding on previous levels.

### Participants

The participants (*N*=10) were recruited from units in the Psychiatric Special Health Care Service in the southern part of Norway. The sample was purposeful, i.e., mothers who experienced the phenomenon of being a mother, suffering from mental illness, and receiving care from professionals in psychiatric specialist health care contexts. Inclusion criteria were Norwegian-speaking mothers, having children between 0 and 18 years, admitted to a hospital unit. The participants’ ages ranged from 28 to 53 years, and the majority lived with a partner. They had been diagnosed with depression, anxiety, bipolar disorder and ADHD and were undergoing individual or group therapy at the time of the interviews. In total, they had 24 children ranging in age from 8 months to 26 years. Exclusion criteria were being committed and actively psychotic or suicidal in the previous 6 months or under the influence of alcohol or drugs at the time of the interview. They were informed about the study and invited to participate by their therapist, and after agreeing to take part, they were contacted by the first author who made an appointment for an interview.

### Data collection

The data was collected over a 3-month period in spring 2010, and took place in the participants’ homes or an office at the clinic in accordance with the wishes of the participant. The opening question was: *What are your experiences of being a mother and struggling with mental illness?* The interview was guided by the following themes: experiences of motherhood, experiences of receiving help from health care professionals in the psychiatric context, and their thoughts about their lives and the future.

This study concerns experiences of being in care provided in a psychiatric context.

An open dialogue with open-ended question was chosen in order to allow the participants to freely express their experiences in as much detail as they wished. Follow up question was posed in order to deepen their narratives, and to avoid ambiguity or misinterpretation. The interviews lasted between 60 and 90 min, were conducted and transcribed verbatim by the first author, and resulted in approximately 482 pages of text.

### Ethical considerations

Ethical approval for the study was granted by The Regional Committee for Medical Research Ethics of South Norway (No. 2009/124) and the Norwegian Social Science Data Services (No. 18667). The study was performed in accordance with The International Counsil of Nurses Code of Ethihs (2012), The National Advisory Board on Research Ethics in Finland (2012), and The World Medical Association Declaration of Helsinki (2013). The potential participants received verbal and written information from their therapist who requested their permission for the researcher to contact them. All agreed to participate and gave their written consent. They were informed of the possibility to withdraw from the study without any consequences. The participants’ identity was protected by paraphrasing their statements while retaining the meaning. The caritas motive in caring science, i.e., ethics had precedence over ontology and epistemology in every part of the study, i.e., love, charity, respect and reverence for human dignity (Eriksson, Lindström, Matilainen, & Lindholm, 2007).

## Result of the interpretation on the contextual level

The interpretation on the contextual level is based on the rational level, i.e., the interview text, and the researchers’ pre-understanding. The contextual level describes and clarifies the overall pattern exhibited by the situation of being in care provided in a psychiatric care context. It describes an understanding of the experiences seen from the mothers’ perspectives.

### Dare I say it? The anxiety of disclose their inner world

The mothers spoke about their feelings of relief at having an opportunity to talk about their anxiety and uneasiness. Narrating their story made them more familiar with themselves and their life; nevertheless, they experienced a feeling of coming to a standstill in life. They learned practices and techniques to master their confusing feelings, so on the outside they would exhibit an appearance of being well. Inside, however, they still experienced a nagging uneasiness as if having something on one's conscience. Their daily lives improved, but the feeling of having something on one's mind was still disturbing.

I was allowed to talk about myself and what I find difficult; about not only the anxiety, but also now, I have a feeling of being at a standstill in life, and the treatment don't make me better. Now I will stop going there, just keep on working with the techniques I learned.

The mothers understood that they were in care for their own sake and not also because they were mothers. They tried to talk about their worries and guilty conscience pertaining to their children, and they received advice and guidance. However, they still experienced a gnawing doubt related to their lives as mothers, which they did not dare to talk about it in the care situations.

I'm mainly there to receive treatment for myself, but I'm more worried about my children. I'm afraid that if I raise my concerns about my children it will be an extra burden on the therapist, because on the one occasion that I mentioned it, he just said that I could bring the children and we could talk to a family therapist –so there are several opportunities available, but ….

The mothers talked about their sense of not having the opportunity to speak openly and honestly about their inner thoughts and feelings, their inner worlds with confusing feelings of guilt and shame were sensed as something unmentionable. When their experiences of feelings and thoughts were considered as a diagnosis it felt strange, and they feared that the diagnosis would come to define them as mothers. They felt they were at risk of losing their children, so they concealed their inner feelings and thoughts by withdrawing into silence and/or by demonstrating their appropriateness as mothers.

I feel that the diagnosis is strange. Being labelled with the diagnosis implies that I will become like the diagnosis, … not that they will necessarily take my children away from me but will say that I'm not capable as a mother or ….

They were in a situation where they, on the one hand, were not allowed to talk about their inner feelings even if they wanted to, and, on the other hand, sensed a risk of being condemned as mothers if they revealed their inner feelings, which meant that it seemed better to appear as unmoved and unburdened. They talked about being trapped in a precarious situation that was frightening and burdensome, and after a while they just wanted to escape.

### The result of the interpretation on the existential level

The interpretation on the existential level is based on the data and the researchers’ pre-understanding, previous research, and the theoretical perspective. The interpretation on this level searches for an understanding of the human being's existential dimension. It is deductive and takes as its point of departure the researchers’ understanding of the theoretical perspective in order to interpret and understand the mothers’ existential world of being in a care provided in a psychiatric care context. Through dialectical reflection on and questioning of the relationship between the caring science theory and the interview text the meaning of the mothers’ experiences related to the existential dimension in life emerged as the following theme: *Living between the silent mask and the beating heart*.

### Living between the silent mask and the beating heart

When suffering distress, the human being is challenged by the fundamental existential question in life. People, when faced with illness and suffering, first and foremost try to understand themselves and their lives. When one's own actions are insufficient to relieve suffering, one seeks the fellowship of other people. Seeking refuge becomes an act of liberation where an encounter with someone who listens can help individuals to lower their mask. The encounter becomes a space for release where the mothers can step forward in calm and tranquility and reveal the burden of their life, in order to find their own way out of themselves and back into the spirit of communion with their inner selves and their children.

In the mothers’ intense and heartfelt longing to find their inner strength, they search for care where they can confidently grasp the lifeline thrown by someone who hears their beating heart. Sharing the burden with another can bring about reconciliation with life and destiny, but also the risk of leaving oneself open to criticism. The invitation and its welcome form the foundation of the mothers’ experiences of being allowed to be the persons they are. When banishing feelings related to motherhood from the care relationship, the mothers are forced to dissociate themselves from their inner essence. When the life-giving creative process in health is reduced to polite comments and techniques, the mothers’ existential freedom to find space to recapture their living space is reduced.

I feel I have much at heart, but when I arrive he asks me about how I have been since the last time, and continues with that, including techniques and exercises and I have no opportunity to say what I was going to say.

When one's inner life is dominated by suffering, it feels as if the whole of life has ground to a halt, but the human being's inner longing and desire remain the same. Following one's inner longing and desire are deeply rooted in the human being, in spite of the most humiliating situations. It includes the inner freedom to drop the silent mask of suffering and create oneself according to one's inner values as demonstrated by the quotation below.

If I have made it sound better than it is, then it's not the truth, because he won't know if he doesn't go straight to the source. If I describe myself in better terms than I am, what he says will not be based on the reality. Therefore, it's very difficult to deal with such things alone.

Care that does not have the capability or capacity to look over and into the mothers’ existence as a mother increases the fear of being judged. When not giving an opportunity to reveal their inner feelings in the caring relationship, they are forced to renounce their inner world. The possibilities for a mutual understanding are rejected in favor of an assessment that is more related to exterior demands than to the mothers’ existence and true experiences. They conceal their feelings by putting on a mask of silence in which they find protection, while at the same time making themselves invisible and unreceptive.

It has something to do with the fear of not being acknowledged as the person I am, so I conceal my feelings. On my way there, I sometimes cry and think about what I really want to say, but when I arrive, I pull myself together. I put my feelings to the side, despite the fact that they are the reason I'm going there. I'm probably afraid of being labelled a lunatic and then they will take my children away ….

The insight about their responsibility as a mother caused them to search for care in the hope of finding a way both into and out of themselves. Turning towards oneself presupposes someone who receives, recognizes the thoughts and feelings as genuine and simultaneously dares to challenge them. The experiences of concealing themselves lead to a fundamental suffering due to indignities and create the basis of how they view themselves as a mother in the care of their children.

## The result of the interpretation on the ontological level

The interpretation on the ontological level seeks to understand the universal and ontological reality of being a patient in care provided in a psychiatric context. The interpretation on this level is based on previous interpretations and understanding, while at the same time being anchored in theoretically charged empirical material. In order to achieve a deeper understanding the philosophy of Levinas (1990, 1992, 2013) is used in addition to the theory of caritative caring (Eriksson, 1990, 1997a, b, 2000, 2001, 2003, 2006, 2007; Lindström et al., 2010).

Based on the empirical material, i.e., the rational level, and interpretations on the contextual and the existential levels, the following hypothesis emerged: Being a mother and receiving care provided in psychiatric care contexts means struggling between the responsibility inherent in their parenting and the fear of condemnation. In the interpretation process on the ontological level, a new hypothesis was formulated: Being in care as patient and parent means struggling to restore one's responsibility as a human being.

The image of parenthood can be viewed as an inherent unconditional humane mission, in the shelter of which the human being lives and acts. The mother's feelings of relief in caring reflect her longings and struggle to return to the bond of responsibility inherent in her parenthood. The responsibility is related to parents’ fundamental knowledge about themselves as a caring human being, which is sensed in the beating heart and cannot be abandoned (cf. Levinas, 2013).

According to Levinas (2013), responsibility is a demand from the Other, the demand to take care of and protect, hence the mother's responsibility is in response to the appeal from the child. The mother is profoundly touched by the child's vulnerability and the mutual dependence she cannot abandon. This is an image of natural mutual agreement between human beings, where the responsibility for the fellow human constitutes the human being (cf. Levinas, 2013). In concrete terms, it is an encounter between a parent and a carer, where the former recognizes the fundamental responsibility in the relationship with the child. At the same time, the encounter represents an appeal for help in order to restore the responsibility inherent in the patient. Here, the ethos of caritative caring emerges as both the starting point and a possibility condition for understanding one's being in the world (cf. Kasén, 2002; Levinas, 2013). In this perspective, the mother enters into the care where the expectation of their own possibility condition includes the same expectation for their children. Parents’ responsibility goes beyond the here and now, beyond the mother as “herself” and into a future in which the mother herself is not a part, but nevertheless considers as her own (cf. Levinas, 1990, 1992, 2013). In this perspective, the mothers step forward as “a fighter and guardian of life.” When their appeal is overlooked or ignored and the response involves the use of power/authority, the patients are forced to resign themselves to the care and the carer's view and concepts (cf. Eriksson, 1997a; Levinas, 2013). In their vulnerability, the mothers do not manage to resist, and in the struggle to restore responsibility, they have to protect themselves and the children by concealing their faces behind a mask. The irresistible and unutterable in caring is pushed aside in favor of that which is already understood.

Caring acts are concrete and lead to a sense of being cared for and protected (Eriksson, 1997b). Lassenius (2005) termed this “the Common Land” which denotes the space in which the mutual creation of doing, being and becoming in health takes place. In caring, mutual creation is the main reason and precondition for the patient stepping forward in their differentness. In order to tend, play and learn it is essential that the patient feels free to act, be and become (Eriksson, 1997b; Kasén, 2002; Lassenius, 2005). Stepping forward in caritative caring presupposes freedom, confidentiality and compassionate listening, which encourages the patient to search for the voice of their heart (cf. Koskinen, 2011). In a caritative caring context the patients may be enabled to meet in unmasked openness and give their conflicting and ambivalent feelings a voice. In a care setting that ignores the drama of their suffering, they are obliged to contain and conceal their lived experiences (cf. Wiklund, 2000). Human beings create themselves and others by means of mutual interplay and mothers bring their creativity in parenthood into the care, even if it is silent and invisible (cf. Lindberg, 2013). The space for creative interplay will be filled by fear in care where the tacit is not heard and that which is absent is not seen (cf. Lassenius, 2005; Lindberg, 2013). When pleasurable play that should provide space for rest and recreation is transformed into exercises and techniques, the joy of life is smothered. The beating heart must be controlled and concealed behind a stiff mask in order to hide the suffering soul. The faces of the patients no longer speak the language of their true heart and they try to appear unburdened out of fear of proving the prejudices correct. This image of the mothers’ experiences of coming to a standstill in life can be understood as being frozen between their inner responsibility and the fear of being the parent they do not want to be.

Silence is the sound of caritative caring, a silent floating voice that conveys a promise of sensitivity, openness and protection (Eriksson, 1997b). In the echo of the child's demand, the mothers experience the intense call of their heart, for which they seek resonance in care (cf. Levinas, 2013). Awareness of the mothers’ responsibility in caritative caring allows the mothers to enter into pleasurable and creative interplay, and thus surrender their vulnerability. Surrender can lead to a process of reconciliation involving a new view of and openness to parenthood, hence openness to the innermost room in which fundamental values reside, i.e., ethos (cf. Eriksson, 2003). When mothers enter their innermost room, it can enable them to take a new direction guided by the responsibility. Caritative caring that confirm the parents’ suffering and reality, understands and dares to challenge their feelings by ask what is in the heart of hearts enable them to live and realize their aspirations based on the responsibility inherent in the human being.

## Implication for clinical practice

Each encounter between a parent and the carer is considered decisive for how the former experience themselves as a parent. Therefore, it is important not only have knowledge of symptoms, diagnosis and treatment methods, but deeply and trustworthy listen to and understand the patient's fundamental responsibility inherent in the human being's existential and ontological dimensions. There is a need for a more distinctly humanistic and dynamic way of thinking and understanding in the psychiatric care context. The care has to be provided as caritative, in which love, compassion, and mercy enable the carer to listen and ask the questions towards the silent voice from the parents’ heart of hearts. Through carefully listening both the mother and the carer might learn something new, or at least realize that there is always something that cannot be completely understood. The basis of every encounter in the clinical practice is respect and humility for the individual and its uniqueness, and simultaneously being aware of the common and mutual space of existence. When parents are given the opportunity to place their trust in someone, they get an opportunity to straighten their attention to their inner demand and unveil the language of their heart of hearts, which might lead to alleviate their suffering.

## Methodological considerations

Hermeneutic interpretation is based on three quality criteria: awareness of one's pre-understanding, the theoretical perspective and the study's inner logical reasoning (Gadamer, 2006; Ödman, 2004, 2007), of which the first two guided the interpretation process. An ongoing dialogue with research colleagues and supervisors challenged our pre-understandings and led to self-reflection. Caring science and hermeneutic epistemology constituted the ontological foundation and methodology of the study, thereby contributing to the hermeneutic interplay between the interpreter, chosen theory and patient experiences (Gadamer, 2006; Ödman, 2004). Hence, the limitations are inherent in the methodology and the theoretical perspective, as well as the researcher. According to the caring science understanding of the human being in an ontological context, there is always something in the human being's life that is unobtainable (Lindström et al., 2010). The human being can hence not be comprehensively grasped; there is always something in her inner essence that can never be understood. The prerequisite for a hermeneutic interpretation is the researcher's pre-understanding. The interpretation is hence not without influence by the researcher's subjectivity. Accordingly, the interpretation includes misunderstandings, attitudes, and habits of interpretation that are not immediately visible or conscious. Vattimo (1997) claims that truth in hermeneutic interpretation is not consistence between the statement and the subject matter, but to what degree the researcher makes diversities and disagreements visible. For Gadamer (2006) the truth is related to experiences that place preceding understanding of the subject matter into a new perspective. The understanding has to be viewed in light of the extent to which it reveals something essential that was previously hidden or emerged differently (Gadamer, 2006). In the abduction phase, the text was decontextualized and placed in dialogue with theory, thus reducing the influence of the pre-understanding.

The description of the different levels of interpretation reveals the interpretation process. Its validity depends on the extent to which the parts illuminate the whole and vice versa. The reciprocity of the epistemology, the theoretical foundation and the quotations may strengthen both the study's inner logic and the empirical foundation. Although the chosen perspective might limit other possible interpretations, it generated a deeper understanding of the perspective of being a mother when being in psychiatric care, thus capturing some of the fundamental ontological life conditions of patients in care.
